# Activation of the Nrf2 Pathway by Sulforaphane Improves Hypoglycaemia-Induced Cognitive Impairment in a Rodent Model of Type 1 Diabetes

**DOI:** 10.3390/antiox14030308

**Published:** 2025-03-04

**Authors:** Heather J. Merchant, Calum Forteath, Jennifer R. Gallagher, Albena T. Dinkova-Kostova, Michael L. J. Ashford, Rory J. McCrimmon, Alison D. McNeilly

**Affiliations:** 1 Division of Diabetes, Endocrinology and Reproductive Biology, School of Medicine, University of Dundee, Dundee DD1 4HN, UK; hmerchant001@dundee.ac.uk (H.J.M.); c.forteath@dundee.ac.uk (C.F.); m.l.j.ashford@dundee.ac.uk (M.L.J.A.); r.mccrimmon@dundee.ac.uk (R.J.M.); 2 School of Life Science, University of Dundee, Dundee DD1 4HN, UK; j.z.gallagher@dundee.ac.uk; 3 Division of Cancer Research, School of Medicine, University of Dundee, Dundee DD1 4HN, UK; a.dinkovakostova@dundee.ac.uk

**Keywords:** diabetes, oxidative stress, Nrf2, cognition, hypoglycaemia

## Abstract

In diabetes, chronic hyperglycaemia leads to cognitive impairment, neurodegeneration and dementia. In a rodent model of streptozotocin (STZ)-induced type 1 diabetes (STZ-T1D), we previously demonstrated that recurrent hypoglycaemia (RH) further exacerbates this process through a mechanism involving increased oxidative and inflammatory stress that overwhelms the compensatory activation of the nuclear factor erythroid 2-related factor 2 (Nrf2) antioxidant system, which was insufficient to prevent cognitive impairment. The current study investigated whether the induction of the antioxidant response through pre-treatment with sulforaphane (SFN), a potent Nrf2 inducer, would ameliorate these cognitive deficits. A mouse model of chronic insulin-treated T1D was achieved using STZ (125 mg/kg i.p.) and insulin implants (Linbit^®^). Diabetic and Control (C57BL6/J) mice were randomly allocated to one of the following seven groups: (i) Control, (ii) STZ-T1D, (iii) Control + RH, (iv) STZ-T1D + RH, (v) Control + RH + SFN, (vi) STZ-T1D + RH + SFN or (vii) STZ-T1D + SFN, and subjected to insulin-induced hypoglycaemia (three episodes per week for four weeks). SFN (50 mg/kg i.p.) or a vehicle (0.1% DMSO/PBS i.p.) were administered 24 h before each hypoglycaemic episode. Cognition was assessed with the Novel Object Recognition (NOR) and spontaneous alternation (SA) tasks. SFN significantly improved the cognitive performance in the 24-h NOR and SA tasks in the STZ-T1D + RH groups. These improvements were absent in the Control or Nrf2-null mice receiving SFN. These studies show, for the first time, that the pharmacological activation of the Nrf2 antioxidant pathway may provide a novel therapeutic target for treating cognitive impairment associated with RH in T1D.

## 1. Introduction

A significant concern for people with type 1 diabetes (T1D) and their carers is whether recurrent hypoglycaemia has long-term consequences on brain function [1,2]. Animal studies have demonstrated that acute severe hypoglycaemia (blood glucose < 1.1 mmol/L) reproducibly induces neuronal damage, specifically within the cortex and hippocampus [3,4]. However, profound hypoglycaemia of this severity is rare in clinical practice. Epidemiological studies of cognitive impairment in people with diabetes have proven inconclusive [5,6], primarily due to the difficulty in accurately recording all but severe hypoglycaemia and the long follow-up duration required to reveal a significant cognitive change in humans. Evidence from the Diabetes Control and Complications Trial (DCCT) and its follow-up, the Epidemiology of Diabetes Interventions and Complications (EDC) study, however, report a more rapid decline in cognitive function in those with a higher HbA1c (indicative of poor glucose control) and exposure to severe hypoglycaemia [7]. In a recent rodent study, we replicated these findings by demonstrating that recurrent hypoglycaemia (RH) in a mouse model of streptozotocin-induced type 1 diabetes (STZ-T1D) produced deficits in learning, memory and behaviour through a mechanism linked to oxidative damage and inflammation in the hippocampus [8]. We further identified that the transcription factor nuclear factor erythroid 2-related factor 2 (Nrf2) was critical in mediating protection against oxidative damage induced in response to RH in non-diabetic animals. However, the Nrf2-initiated response was insufficient to prevent cognitive impairment in T1D.

Consequently, this current study investigated whether the pharmacological enhancement of the endogenous Nrf2 pathway via the administration of the potent Nrf2 inducer sulforaphane (SFN) could offer protection and minimise the impact of RH on cognitive function in our mouse model of STZ-T1D. Phytochemicals like SFN alter this complex through adduct formation with specific Keap1 cysteine residues [9]. This modification of Keap1 leads to the stabilisation of Nrf2 and the subsequent translocation to the nucleus, where it binds to antioxidant response elements within the promoter region of an array of genes encoding proteins involved in detoxification reactions and antioxidant responses [10,11]. Most of the research involving SFN targeting Keap1-Nrf2 has been in the field of cancer, but studies in models of neurological conditions and diabetes are emerging [12,13,14]. Indeed, SFN is an effective and safe chemoprotective agent, with over 61 clinical trials currently registered. To our knowledge, this is the first study to investigate the potential therapeutic efficacy of SFN on cognitive function in T1D. 

## 2. Materials and Methods

### 2.1. Experimental Animals 

Male C57BL6/J (wild type, WT; 10–12 weeks) mice (Charles River Laboratories, UK) and *Nrf2* knockout (KO) mice (initially provided by Yamamoto) were used [15]. The generation and genotyping of Nrf2-KO mice have been described previously [16]. Animals were group-housed and maintained on a 12:12 h light/dark cycle. Drinking water and standard laboratory chow (RM1, SDS diets, UK) were available ad libitum. All of the animal procedures were approved by the University of Dundee Ethical Review Process and performed following UK Home Office regulations (Project Licence PE82c1898).

### 2.2. Experimental Groups

Hyperglycaemia was induced in male C57BL6/J mice with a single injection of streptozotocin (STZ (Merck); 125 mg/kg i.p., max volume 5 mL/kg), with the WT Control mice injected with citrate acid buffer (i.p.). The health and well-being of the STZ-treated animals were maintained via a Linbit^®^ insulin implant (LinShin, Scarborough, ON, Canada), as described previously [8]. Subsequently, the mice were randomly assigned to one of the following seven experimental groups: (i) Control, (ii) STZ-T1D, (iii) Control + RH, (iv) STZ-T1D + RH, (v) Control + RH + SFN, (vi) STZ-T1D + RH + SFN or (vii) STZ-T1D + SFN The experimental plan and sample sizes were calculated following ARRIVE 2.0 guidelines. Group sizes are stipulated in the figure legends, with a minimum of 8 animals in all C57BL6/J groups. Sample sizes were based upon published protocols [8]. Assuming a confidence level of 95%, that is, a confidence level of 1.1 (the SD of average hypoglycaemia in STZ-treated animals), to detect a significant difference between euglycaemic and hyperglycaemic groups, a minimum of 10–12 animals per group were required.

Similarly, male Nrf2-null (KO) mice were randomly allocated to the following four experimental groups: (i) KO, (ii) KO + RH, (iii) KO + RH + SFN or (iv) KO + SFN (*n* = 6–8/group). Nrf2-KO animals were used as a negative control to enable us to determine the specificity of any SFN-mediated changes.

### 2.3. Recurrent Hypoglycaemia

See Supplementary Figure S1 for an experimental timeline. The mice were subjected to 12 episodes of insulin-induced hypoglycaemia (1–4 U/kg s.c., max volume 5 mL/kg Actrapid, Novo Nordisk Ltd., target glucose 2.8 mmol/L; 3 per week for four weeks), as described previously [8]. Blood glucose was measured from the tail vein using a handheld glucose monitor (Accu-check, Roche, Burgess Hill, UK). Hypoglycaemia was maintained for 2 h, and the animals were returned to euglycaemia with food. Control (non-RH) animals were fasted and given volume-matched saline. No animals suffered fits or seizures due to severe hypoglycaemia.

### 2.4. Administration of Sulforaphane (SFN)

Sulforaphane (*D*, *L*-sulforaphane CAS 4478-93-7, Calbiochem, UK) was prepared in DMSO (Sigma-Aldrich), diluted in sterile saline to a DMSO concentration of 0.1% (*v*/*v*) and administered 24 h before each hypoglycaemic episode at a dose of 50 mg/kg, i.p., max volume 5 mL/kg, based upon published literature [12,17,18]. This time point was selected to ensure sufficient time for the transcription and activation of Nrf2 target genes. Control animals received saline (equivalent weight/volume, i.p.).

### 2.5. Behavioural Procedures

Cognition was assessed using the Novel Object Recognition (NOR) task, a hippocampal-mediated task based on rodents’ natural tendency to seek novelty [19]. A discrimination index (D3) (total time spent exploring a novel object/total time spent on a familiar object) provided a numerical measure of short- (10 min) or long (24 h)-term memory. The spontaneous alternation (SA) task assessed spatial working memory [20]. Memory performance was the percentage of 4/5 alternations, with an alternation counted when all four arms of the closed arm plus the maze were visited within five arm choices. The level of chance in this test was 44% [21]. All of the testing was performed in the morning between 0800 and 1100 h, with the first behavioural test taking place 3 days after the last hypoglycaemic episode (see Supplementary Figure S1 for an experimental timeline). For the NOR task, any animal that failed to explore the objects for a minimum of 15 s during the sample phase was excluded from further analysis.

### 2.6. Biochemical Analyses

After the last behavioural test, animals were killed humanely; then, brain tissue was dissected into specific regions and flash-frozen in liquid nitrogen for the subsequent biochemical analyses. 

### 2.7. Lipid Peroxidation and Protein Carbonylation

The frozen hippocampal tissue was powdered in liquid nitrogen to ensure a homogeneous tissue fraction was used for each biochemical assay. The protein concentration was determined using a Bradford protein assay, and samples (50 µg of protein) were assayed in duplicate. Hippocampal lipid peroxidation products were measured using the thiobarbituric acid-reactive substances (TBARS) assay as described [22]. Hippocampal carbonylated protein levels were measured by ELISA (Cayman Chemical, Ann Arbor, MI, USA) according to the manufacturer’s guidelines.

### 2.8. Inflammatory Protein Array

Hippocampal levels of inflammatory cytokines (IFNg, IL12p-70, IL-2, IL-4, IL-5, IL-6, IL-10, TNFα, IL1β and KC/GRO (CXCL1)) were measured using the V-plex Proinflammatory Panel (mouse; Meso-Scale Discovery, MSD, London, UK) following the manufacturers’ guidelines. Hippocampal samples (50 μg of protein) were assayed in duplicate. 

### 2.9. RNA Extraction and PCR

Total RNA was extracted from the hippocampus using TRIzol reagent (Invitrogen). The integrity and RNA concentration of the samples were assessed using a spectrophotometer (Nanodrop 2000, Thermo, Waltham, MA, USA), with an rRNA ratio of between 1.8 and 2, corresponding to the acceptable integrity and minimal content of RNA lysing impurities. Reverse transcription (1 ng of RNA) was performed using the SuperScript^®^ III First-Strand Synthesis System for Reverse Transcription (RT) (Invitrogen, Paisley, UK). Real-time PCR was performed using TaqMan gene expression assays for Nrf2 and associated target genes (Applied Biosystems; Supplementary Table S2). All of the experiments were performed using the most up-to-date guidelines for quantitative PCR (qPCR) [23]. All of the samples were run in triplicate, and the mRNA levels were assessed relative to the housekeeping genes cyclophilin and actin using the comparative Ct(−∆∆Ct) method. The mRNA levels of these housekeeping genes did not change with the treatment. Data are expressed as the fold-change relative to the appropriate control for each group, i.e., Control, T1D or Nrf2-KO.

### 2.10. Statistical Analysis

Data were analysed using SPSS version 21 (SPSS, Chicago, IL, USA). Multivariate ANOVA was used to compare the groups with the treatment (SFN) and RH as between-subject variables. When the data did not follow a normal distribution, as determined by the Shapiro–Wilk Test, a non-parametric Kruskal–Wallis analysis was performed. Nrf2-KO mice were assessed as an independent group, as they were primarily used to demonstrate the specificity of SFN and not the impact of T1D per se. For the post hoc analysis, the Tukey multiple comparisons test (parametric) and Bonferroni (non-parametric) test were used. Data visualisation was performed using GraphPad Prism (Version 9.4.1). Data are expressed as mean ± SEM. Statistical significance was set at *p* < 0.05.

### 2.11. Data and Resource Availability

The data sets generated and analysed during the current study are available from the corresponding author upon reasonable request.

## 3. Results

### 3.1. Sulforaphane Had No Impact on Body Weight or Glycaemic Control

As anticipated, the STZ-T1D animals were lighter (the effect of the STZ, *p* < 0.01) and had significantly higher fasting glucose levels than the non-diabetic Control (WT) animals (the effect of the STZ, *p* < 0.01). The Nrf2-KO animals were markedly heavier than the Control and T1D animals (the effect of the genotype, *p* < 0.01). Recurrent hypoglycaemia (RH) had no impact on body weight in any group (*p* = ns), and there was no difference in the glucose level of hypoglycaemia achieved between groups (the effect of the Hypo; *p* = ns). Likewise, the SFN had no impact on body weight, blood glucose levels or the level of hypoglycaemia achieved in any group (*p* = ns for all; Supplementary Table S1).

### 3.2. Sulforaphane Improves Spatial and Long-Term Working Memory in Diabetic Animals

**Figure 1:**
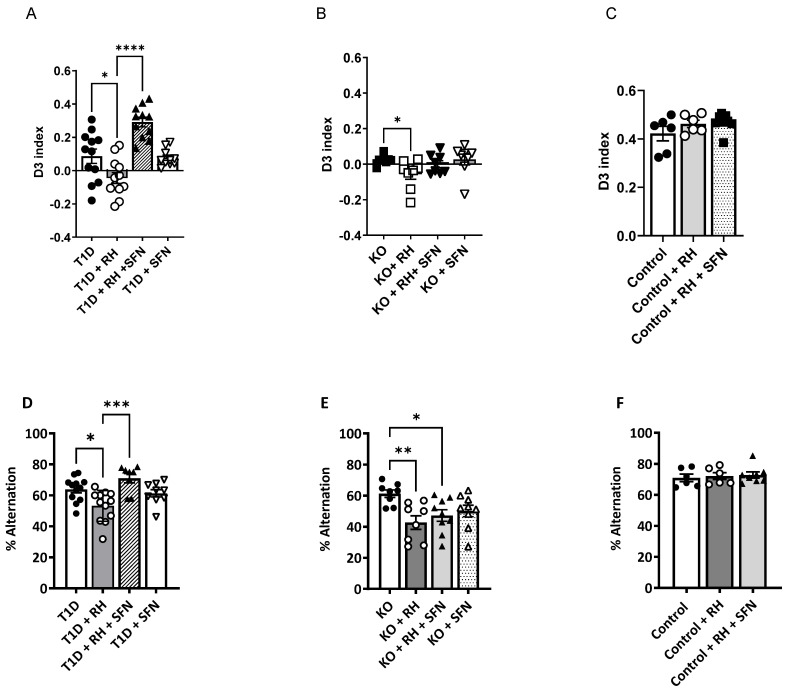
Sulforaphane improves the cognitive performance in the 24-h Novel Object Recognition (NOR) and spontaneous alternation (SA) tasks in the STZ-T1D animals exposed to RH: (**A**): The D3 index, which was markedly below 0.39, demonstrated that the T1D mice were impaired when tested in the 24-h NOR task (*p* < 0.05). This deficit was exacerbated in the STZ-T1D animals following the RH (*p* < 0.05) and partially rescued following the SFN administration. (**B**): The performance of the Nrf2-KO mice was impaired in the 24-h NOR task for all of the treatment regimens (*p* < 0.001). This impairment was exacerbated following the RH (*p* < 0.05) (**C**): There was no impact of the RH or SFN in the Control animals on the performance in the NOR task, with all of the animals showing a preference for the novel object (D3 ≥ 0.4). (**D**). The mean 4/5 alternation performance on a closed-arm-plus-maze, expressed as a percentage of possible alternations, was reduced in the STZ-T1D animals (*p* < 0.05) and further impaired following the RH when compared to the non-diabetic Control mice (*p* < 0.01) [8]. Supplementation with SFN improved cognitive function in the STZ-T1D + RH animals to a level comparable with the non-diabetic Control animals. (**E**): The performance in the SA task was impaired in all Nrf2-KO mice (*p* < 0.05) compared to the non-diabetic Control animals. Exposure to RH further impeded the performance compared to their non-RH Nrf2-KO counterparts (*p* < 0.01), with the SFN supplementation being ineffective. (**F**)*:* RH or SFN had no impact on the performance in the SA task (*p =* ns; *n* = 6–12/group). The results represent the mean values ± SEM. Data were analysed by two-way ANOVA, with the RH and SFN as between-subject factors, followed by the Tukey post hoc test. * *p* < 0.05; ** *p* < 0.01; *** *p* < 0.001, **** *p* < 0.0001. (**A**,**D**) Filled circles = T1D, open circles = T1D + RH, filled triangles = T1D + RH + SFN, open triangles = T1D + SFN; (**B**,**E**); Filled circles = Nrf2 KO, open circles = Nrf2 KO + RH, filled triangles = Nrf2 KO + RH + SFN, open triangles = Nrf2 KO + SFN. (**C**,**F**) Filled circles = Control, open circles = Control + RH, filled triangles = Control + RH + SFN.

In keeping with previous findings [8], all of the animals were able to complete the Novel Object Recognition (NOR) task with an inter-trial interval of 10 min, indicating short-term memory was intact, irrespective of the diabetes or Nrf2 status (D3 index ≥ 0.3; inter-trial interval 10 min). After 24 h, long-term memory was impaired in the STZ-T1D rodents relative to the Control (D3 index; Control 0.39 ± 0.12 vs. STZ-T1D + RH − 0.05 ± 0.03, *p* < 0.01; Figure 1A,C), an effect exacerbated by the RH (the effect of the T1D, *p* < 0.01; T1D × RH, *p* < 0.05; Figure 1A). The administration of SFN partially rescued this deficit, although not to the competency of the non-diabetic Controls (D3 index; Control 0.39 ± 0.12 vs. STZ-T1D + RH + SFN 0.29 ± 0.03, *p* = 0.05; Figure 1C). Animals lacking functional Nrf2 (Nrf2-KO) were significantly impaired in the NOR task when compared to the Control animals (the effect of the genotype, *p* < 0.01), showing no preference for either object (D3 index = 0). This defect was exacerbated following exposure to the RH (the effect of the RH *p* < 0.05; Figure 1B). There was no improvement in cognition in the Nrf2-KO animals receiving the SFN (Figure 1B; *p* = ns). Likewise, the SFN did not impact the performance in the Control + RH animals (Figure 1C; *p* = 0.27). Importantly, all of the animals explored the novel object for at least 20 s within a 10-min testing period [24]. There was no difference in the total exploration time in any of the groups (Supplementary Figure S2A–C), demonstrating that the deficits detected in the diabetic animals were not due to a lack of locomotor ability or motivation.

Spatial working memory, tested by the spontaneous alternation (SA) task, was impaired in all of the STZ-T1D animals, with a significantly reduced % alternation compared to the non-diabetic Controls (Control 70.9 ± 2.4 vs. STZ-T1D 62.9 ± 2.2; effect of T1D, *p* < 0.01; Figure 1D), thus corroborating our previous findings [8]. These cognitive defects were augmented further in the STZ-T1D animals exposed to the RH (T1D × Hypo, *p* < 0.01). The SFN improved the working memory in the STZ-T1D + RH animals (Hypo × SFN, *p* < 0.05; Figure 1D) but did not improve the working memory in the STZ-T1D-alone animals (STZ-T1D × SFN, *p* = 0.06; Figure 1D). The improvement in performance in the STZ-T1D + RH animals following the SFN treatment was not due to an increase in the total arm entries, which were comparable between all of the groups (Supplementary Figure S2E; *p* = 0.174). In keeping with the NOR findings, the RH impaired the spatial working memory in the Nrf2-KO animals (the effect of the Hypo, *p* < 0.01; Figure 1F); however, the SFN did not improve the performance (*p* = ns; Figure 1F). Importantly, the spatial working memory was impaired in all groups of Nrf2-KO mice relative to the non-diabetic Controls (the effect of the genotype, *p* < 0.01). The RH or SFN did not impact the % alternation (Figure 1D; *p* = 0.84) or total entries (Supplementary Figure S2D; *p* = 0.30) in the Control animals.

### 3.3. Sulforaphane-Mediated Induction of the Nrf2 Pathway Induces Antioxidant Defence Associated with STZ-T1D and RH

Compared with the STZ-T1D animals, the RH increased the transcript abundance of *Nfe212*, the gene encoding Nrf2, in the hippocampus (the effect of the Hypo, *p* < 0.01), an effect also seen with the SFN (the effect of the SFN, *p* < 0.01) and further augmented in the STZ-T1D + RH + SFN mice (Hypo × SFN, *p* < 0.01; Table 1). Likewise, the mRNA levels for the classical Nrf2 target gene *Nqo1* were elevated following the administration of SFN compared to the untreated STZ-T1D or STZ-T1D + RH animals (the effect of the SFN, *p* < 0.01; Table 1), although *Hmox-1* and *Sod2* were unaffected by these interventions. The transcript abundance of the glutamate–cysteine ligase catalytic subunit (*Gclc*) and glutamate–cysteine ligase regulatory subunit (*Gclm*), which together form the rate-limiting enzyme in glutathione synthesis, were also enhanced in the STZ-T1D + RH animals (the effect of the Hypo, *p* < 0.05) and maintained in the presence of the SFN (the effect of the SFN, *p* < 0.05; Table 1). To determine whether the effects of the SFN were mediated by Nrf2 or due to modifications in alternative pathways, the transcript abundance of the Nrf2 target genes was assessed in the Nrf2-KO mice challenged with the RH and SFN. The RH or SFN did not affect the transcripts measured (Table 1). In keeping with the anticipated effects of the SFN, the mRNA levels of Nrf2 and its target Nqo1 were significantly elevated in the Control animals receiving the SFN (the effect of the SFN, *p* < 0.05; Table 1). Neither the RH nor SFN had any impact on the expression of Hmox-1 or Sod 2 (*p* = ns; Table 1) in the Control animals. However, the expression of both Gclc and Gclm were significantly greater in the Control animals treated with the SFN (the effect of the SFN, *p* < 0.05; Table 1).

### 3.4. Sulforaphane Decreases ROS-Induced Cell Damage Within the Hippocampus

**Figure 2:**
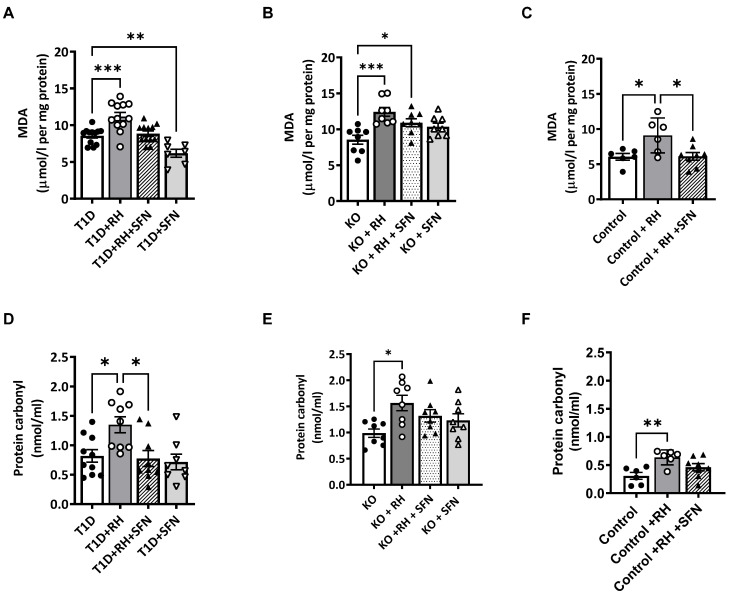
Sulforaphane reduces oxidative stress-induced damage in the hippocampus of STZ-T1D animals exposed to RH. Levels of (**A**): lipid peroxidation (*p* < 0.001) and (**D**): protein carbonylation (*p* < 0.05) were elevated in the STZ-T1D animals following RH. Supplementation of the STZ-T1D + RH mice with the SFN reduced the lipid peroxidation (*p* < 0.01) and protein carbonylation (*p* < 0.05) to T1D levels. Similarly, the RH in the Nrf2-KO animals increased the levels of (**B**): lipid peroxidation (*p* < 0.001) and (**E**): protein carbonylation (*p* < 0.01), which were unaltered following the administration of SFN to Nrf2-KO and Nrf2-KO + RH mice. In the Control animals, the RH increased the levels of (**C**): lipid peroxidation (*p* < 0.05), and the administration of SFN reduced peroxidation to Control levels but had no effect on (**F**): protein carbonylation. Data were analysed by two-way ANOVA with RH and SFN as between-subject factors, followed by the Tukey post hoc test. *n* = 6–12/group. Results represent mean values ± SEM. * *p* < 0.05, ** *p* < 0.01,*** *p* <0.001 vs. appropriate controls (T1D, KO or Control). MDA = malondialdehyde.

Previously, we reported that RH causes Nrf2-dependent redox imbalance, leading to hippocampal oxidative damage and cognitive impairment [8]. Therefore, we investigated the impact of SFN on ROS-induced cellular damage markers, lipid peroxidation and protein carbonylation. The levels of lipid peroxidation were elevated in the STZ-T1D animals following the RH (the effect of the Hypo, *p* < 0.01; Figure 2A), as previously reported [8]. The administration of the SFN to the STZ-T1D + RH mice reduced the lipid peroxidation to T1D levels (Hypo × SFN, *p* = ns). The post hoc analysis revealed that lipid peroxidation was suppressed further by the SFN in the STZ-T1D mice, suggesting that chronic hyperglycaemia induced by STZ-T1D alone is a source of ROS that is sufficient to damage hippocampal lipid membranes (the effect of the SFN, *p* < 0.01; Figure 2A). The RH increased the lipid peroxidation in both the Control and Nrf2-KO mice (the effect of the Hypo, *p* < 0.01 and *p* < 0.05; Figure 2B,C, respectively), whereas the SFN reduced the levels of lipid peroxidation to basal levels in the Control animals (the effect of the SFN, *p* < 0.01; Figure 2C); this was not observed in the Nrf2-KO mice (the effect of the SFN, *p* = ns; Figure 2B). Protein carbonylation, an irreversible post-translational modification in response to elevated levels of reactive oxygen species (ROS), was increased in the STZ-T1D animals following the RH (the effect of the Hypo, *p* < 0.01) and suppressed by the SFN (the effect of the SFN, *p* < 0.01; Figure 2D). The RH also increased the protein carbonylation within the hippocampus of the Nrf2-KO mice (the effect of the Hypo, *p* < 0.05; Figure 2E), an effect that was unaltered following the SFN (the effect of the SFN, *p* = ns). In keeping with the findings of lipid peroxidation, the levels of protein carbonylation in the Control animals were significantly elevated following the RH (the effect of the Hypo, *p* < 0.01; Figure 2F) and reduced with the SFN to Control levels (the effect of the SFN, *p* = 0.08 vs. Control).

### 3.5. Sulforaphane Decreases Inflammation in the Hippocampus of STZ-T1D and RH Mice

Increased systemic and cerebral inflammation are key mediators in the pathophysiological features of diabetes and the associated vascular complications [25]. Furthermore, hypoglycaemia induces a pro-inflammatory response, with elevated levels of IL-1β and TNFα in both clinical and in vivo settings. Thus, we next examined the levels of a panel of inflammatory cytokines in the hippocampus of STZ-T1D and RH mice. In keeping with our previous study, the RH in T1D animals was associated with increased hippocampal levels of the pro-inflammatory cytokines IL-1β and TNFα (Table 2; the effect of the Hypo, *p* < 0.05 and *p* < 0.01, respectively) [8], which were reduced following the treatment with the SFN (Table 2). Additionally, the SFN treatment resulted in an increase in the anti-inflammatory cytokine IL-10 in both the T1D + SFN and T1D + RH + SFN animals (Table 2; the effect of the SFN, *p* < 0.05). Interestingly, the SFN treatment also lowered the hippocampal levels of these pro-inflammatory cytokines in the Nrf2-KO animals, suggesting that the SFN inhibits inflammation through both Nrf2-dependent and independent mechanisms.

## 4. Discussion

In the current study, we demonstrated that the pharmacological activation of the Nrf2 pathway by SFN partially reverses the cognitive impairments associated with RH in STZ-T1D mice. The improvements in cognition were associated with the increased expression of the Nrf2 target genes *Gclc* and *Gclm*, and a reduction in the oxidative damage measured by lipid peroxidation and protein carbonylation. Additionally, the SFN reduced the levels of the pro-inflammatory cytokines IL-1β and TNFα in the hippocampus of the STZ-T1D animals exposed to the RH animals, in keeping with the improvements with the cognitive function observed in this group. Moreover, an increase in the anti-inflammatory protein IL-10 was detected in all of the SFN-treated STZ-T1D mice. Notably, the SFN had no impact on any parameter assessed in the Nrf2-KO mice. Indeed, the Nrf2-deficient animals had impaired recognition memory at baseline, which was augmented following the RH. Furthermore, the exposure of Nrf2-KO mice to the RH resulted in a significant impairment in spatial working memory, which was unaltered following the administration of the SFN. These findings suggest that endogenous Nrf2 activity provides baseline neuroprotection against oxidative stress-induced damage, such as that seen in response to RH, ageing [26] and potentially the chronic hyperglycaemia of diabetes.

Following the treatment of hypoglycaemia in T1D, systemic glucose often increases significantly above normal physiological levels. Glucose reperfusion in end-organs is a significant source of ROS [4,27]. The brain is particularly vulnerable to this due to its dependency on glucose, high metabolic rate and limited antioxidant capacity. In both human and rodent models, the chronic hyperglycaemia of diabetes is associated with an impaired endogenous antioxidant response within key tissues, such as the brain, leading to a redox imbalance [28]. We have previously demonstrated that exposure to chronic hyperglycaemia significantly impairs the ability to cope with a subsequent stressor, e.g., hypoglycaemia [29]. Therefore, it is possible that, in this study, the SFN-mediated activation of the Nrf2 pathway is only protective against acute challenges such as intermittent hypoglycaemia and not chronic hyperglycaemia, which was established before the SFN treatment began. These findings partly explain why the SFN did not affect the cognition in the STZ-T1D animals alone. Indeed, the administration of the SFN increased the transcript levels of Nrf2 and certain Nrf2 target genes, including *Nqo1*, involved in mediating the antioxidant response, and *Gclm* and *Gclc*, the major components of glutamate–cysteine ligase. However, only *Nqo1* expression was synergistically elevated in response to both RH and SFN, with the expression of *Gclc* and *Gclm* increased to a similar degree by the SFN or RH. These findings suggest that the increase in *Nrf2* and *Nqo1* expression in response to RH may primarily represent a natural defence mechanism to combat ROS production in an already stressful environment, such as chronic hyperglycaemia in diabetes. In our model of STZ-induced T1D and recurrent hypoglycaemia (STZ-T1D + RH), the addition of SFN augmented this further, which may explain the partial alleviation of the ROS-mediated damage (lipid peroxidation and protein carbonylation) associated with the episodes of hypoglycaemia.

In contrast, RH and SFN increase the expression of *Gclc* and *Gclm* to a similar degree. One of the most recognised roles of Nrf2 is the maintenance of redox homeostasis, particularly during periods of increased oxidative stress. Thus, the increase in both *Gclc* and *Gclm* is in keeping with this role. However, in response to chronic stress, such as those with poorly controlled type 1 diabetes [30], basal levels of GSH are decreased. Therefore, treatment with SFN may only serve to restore GSH homeostasis, with the system unable to mount a further response when challenged with additional stressors. Thus, acute stress (RH) or controlled (SFN) Nrf2 activation may offer protection, whilst chronic activation (particularly in an autophagy-deficient setting) can be detrimental.

It is important to note the total levels of GSH and the GSSG/GSH ratio were not measured in this study. Likewise, the Nrf2 stability and the levels of Nrf2 target proteins were not assessed in the current study and require further investigation. Furthermore, the positive outcomes related to the SFN were modest, which suggests that chronic diabetes and RH may elicit damage through multiple different pathways. In addition, although SFN is a potent inducer of Nrf2, it has also been shown to modulate numerous proteins that affect various processes, including proliferation, cell cycle, apoptosis and migration through non-Nrf2-dependent mechanisms. Nonetheless, the absence of improvement in cognitive performance in the Nrf2-KO animals strongly suggests that the observed effect of the SFN in the RH is Nrf2-dependent.

Notably, the SFN was able to reduce inflammation in the Nrf2-KO mice, which corroborates previous reports showing that the anti-inflammatory effects of SFN are only partly Nrf2-dependent. When primary murine macrophages were stimulated with IFN-γ and TNF-α, the SFN treatment suppressed the induction of iNOS in both wild-type and Nrf2-KO cells. However, SFN was a less potent inhibitor in the Nrf2-deficient macrophages [31]. The anti-inflammatory properties of Nrf2 are thought to be mediated via several mechanisms, including the transcriptional upregulation of enzymes encoded by Nrf2 target genes [32] and the suppression of genes encoding pro-inflammatory cytokines such as IL-1β and IL-6 [33]. In addition to Nrf2, it has been shown that SFN has anti-inflammatory properties mediated by the suppression of nuclear factor-κapa B (NF-κB), the subsequent downregulation of pro-inflammatory cytokines and mediators [34], and the inhibition of inflammasomes [35] and macrophage migration inhibitory factor (MIF) [36]. Overall, our findings are in keeping with previous studies demonstrating that the anti-inflammatory effects of SFN are mediated via both Nrf2-dependent and non-Nrf2-dependent mechanisms.

An important factor to note is the timing of the SFN administration. In this study, SFN was administered 24 h before each acute hypoglycaemic episode. This time point was chosen to provide sufficient time for the transcription and the subsequent translation of the target proteins. In addition, the average individual on insulin therapy will typically experience symptomatic hypoglycaemia 2–3 times per week [37]. Therefore, this dosing regimen provided the most feasible way of covering both the experimental and clinical exposure to hypoglycaemia. Although SFN has a relatively short half-life of approximately 2.2 h [38], the resulting increase in the expression of Nrf2 target genes and corresponding proteins, including *Nqo1* and the enzymes involved in the biosynthesis of glutathione, remain elevated for several days [39]. Indeed, Bergström et al. proposed that the repeated stimulation of the Nrf2 pathway may provide long-term protection from oxidative damage. Further studies should determine whether the administration immediately before or during recovery from the hypoglycaemic episode, where we predict more damage occurs, would have had a greater effect. Moreover, no improvements in cognition were noted in the T1D + SFN animals, which suggests a preventative protective effect of SFN against RH rather than the recovery of previously induced damage by chronic hyperglycaemia. However, improvements in lipid peroxidation were noted in the T1D + SFN animals, suggesting the recovery of pre-existing damage to some extent. In the wider literature, SFN has been shown to exert both preventative effects [40] and rescue pre-existing damage [18].

A limitation of this study is the use of streptozotocin (STZ) to induce the T1D phenotype. Streptozotocin, a glucosamine–nitrosourea compound derived from *Streptomyces achromogeness*, induces long-term hyperglycaemia and hypoinsulinaemia in rodents via Glut2-mediated uptake into the insulin-producing beta cells, thus selectively destroying the insulin-producing cells. Other models, such as the Non-obese Diabetic (NOD) mouse model, are unsuitable for our experiments due to the requirement for housing in specific pathogen-free environments, which would preclude any cognitive testing and the induction of recurrent hypoglycaemia. Furthermore, only male animals were used in this study, and there is a known sexual dimorphism in Nrf2 expression (which is higher in females than in males) and difference in their responsiveness to treatment. Therefore, it will be important to repeat these studies in female animals. Furthermore, we cannot exclude the possibility that the poor performance observed in the T1D animals was due to anhedonia or disinterest. Due to the hyperphagic nature of T1D rodents, food-motivated tasks, such as the sucrose preference test, frequently employed to assess anhedonia or operant-based tasks, such as the delay-discounting task, are not suitable. Importantly, we did not see any difference in the time taken to make either the first arm entry in the spontaneous alternation task or to interact with objects in the NOR task, suggesting that motivation (or lack) is not the main factor driving the cognitive deficits observed in this study. Finally, a caveat of this study is the rate of sulforaphane metabolism in rodents versus humans, which should be considered for any future clinical intervention study.

In summary, the pharmacological induction of the Nrf2 pathway by SFN offers some protection against RH-induced cognitive dysfunction in diabetes. Nrf2 activators are currently in advanced clinical trials for multiple chronic conditions [41], and dimethyl fumarate and omaveloxolone (RT-408) are clinically used for the management of relapsing–remitting multiple sclerosis [42] and Friedreich’s ataxia [43], respectively, which may provide a novel therapeutic strategy for managing RH-induced cognitive dysfunction in T1D.

## Figures and Tables

**Table 1:** Sulforaphane stimulates the expression of genes involved in mediating antioxidant and redox systems within the hippocampus of T1D animals following recurrent intermittent hypoglycaemia. The mRNA was extracted and processed for the real-time qPCR to evaluate the changes in the gene expression of *Nrf2*, heme oxygenase 1 (*Hmox-1*), NAD(P)H: quinone oxidoreductase (*Nqo1*), superoxide dismutase 2 (*Sod2*), glutamate–cysteine ligase catalytic subunit (*Gclc*) and glutamate–cysteine ligase regulatory subunit (*Gclm*). The results represent the mean values ± SEM relative to each appropriate Control. Data were analysed by two-way ANOVA, with the RH and SFN as between-subject factors, followed by the Tukey post hoc test. * *p* < 0.05 vs. Control (columns 1–3); * *p* < 0.05, ** *p* < 0.01 vs. T1D; ^#^ *p* < 0.05 vs. T1D + RH + SFN (columns 4–7). ND = not determined.

	1	2	3	4	5	6	7	8	9	10	11
	Control	Control + RH	Control + RH + SFN	T1D	T1D + RH	T1D + RH + SFN	T1D + SFN	Nrf2^−/−^	Nrf2^−/−^ + RH	Nrf2^−/−^ + RH + SFN	Nrf2^−/−^ + SFN
*Nrf2*	1.0 ± 0.07	0.96 ± 0.08	1.49 ± 0.15 *	1.0 ± 0.05	1.46 ± 0.02 **^#^	1.82 ± 0.07 **	1.46 ± 0.07 *^#^	ND	ND	ND	ND
*Nqo1*	1.0 ± 0.06	0.95 ± 0.21	1.51 ± 0.13 *	1.0 ± 0.06	1.31 ± 0.09 *^#^	1.87 ± 0.15 **	1.59 ± 0.16 **	1.0 ± 0.06	1.05 ± 0.04	0.92 ± 0.05	1.03 ± 0.04
*Hmox-1*	1.0 ± 0.09	0.96 ± 0.09	0.97 ± 0.06	1.0 ± 0.06	1.34 ± 0.09	1.32 ± 0.08	1.32 ± 0.08	1.0 ± 0.10	1.07 ± 0.12	1.07 ± 0.14	1.14 ± 0.09
*Sod2*	1.0 ± 0.14	0.94 ± 0.06	1.01 ± 0.05	1.0 ± 0.14	0.81 ± 0.04	0.87 ± 0.06	0.82 ± 0.04	1.0 ± 0.10	0.94 ± 0.15	0.80 ± 0.05	1.10 ± 0.09
*Gclc*	1.0 ± 0.05	0.98 ± 0.08	1.48 ± 0.15 *	1.0 ± 0.05	1.43 ± 0.11 *	1.50 ± 0.15 **	1.49 ± 0.09 *	1.0 ± 0.05	1.05 ± 0.04	0.93 ± 0.05	1.04 ± 0.02
*Gclm*	1.0 ± 0.03	0.99 ± 0.04	1.31 ± 0.07 *	1.0 ± 0.10	1.43 ± 0.12 *	1.62 ± 0.11 **	1.31 ± 0.10	1.0 ± 0.05	1.04 ± 0.06	0.94 ± 0.07	0.97 ± 0.04

**Table 2:** The impact of RH and sulforaphane on hippocampal cytokine levels in T1D and Nrf2^−/−^ mice. The levels of several common inflammatory cytokines were measured within hippocampal homogenates by ELISA. IL-4 was below the range of detection across all of the samples; therefore, it has been omitted from the table. The results represent the mean values ± SEM. Data were analysed by two-way ANOVA, with the RH and SFN as between-subject factors, followed by the Tukey post hoc test. * *p* < 0.05, ** *p* < 0.01 vs. T1D (columns 1–4); * *p* < 0.05, ** *p* < 0.01 vs. Nrf2^−/−^ (columns 5–8); ^#^ *p* < 0.05 Nrf2^−/−^ + RH vs. Nrf2^−/−^ + RH + SFN.

	1	2	3	4	5	6	7	8
	T1D	T1D + RH	T1D + RH + SFN	T1D + SFN	Nrf2^−/−^	Nrf2^−/−^+ RH	Nrf2^−/−^+ RH + SFN	Nrf2^−/−^+ SFN
Pro-inflammatory								
*IFNg*	4.77 ± 0.94	4.15 ± 0.66	4.31 ± 0.78	4.15 ± 0.58	5.17 ± 0.44	4.67 ± 0.65	6.61 ± 0.41	6.57 ± 0.69
*IL-12p70*	117 ± 10.2	128 ± 13.2	127 ± 14.4	131 ± 10.1	163 ± 33.2	158 ± 7.27	147 ± 14.5	74.9 ± 14.1
*IL-1β*	2.35 ± 0.51	8.61 ± 1.55 *	6.22 ± 1.23	5.14 ± 1.68	3.42 ± 0.31	4.56 ± 0.25 *	1.20 ± 0.24 ^#^	1.89 ± 0.12
*IL-2*	15.3 ± 1.91	12.3 ± 1.19	13.5 ± 0.78	14.9 ± 1.74	12.5 ± 1.40	12.7 ± 1.06	18.2 ± 1.88	20.1 ± 1.46
*IL-5*	47.8 ± 3.87	29.1 ± 1.96 **	42.3 ± 4.71	42.2 ± 3.71	34.3 ± 1.97	58.5 ± 6.12 **	51.9 ± 7.47	53.9 ± 4.93
*KC/GRO*	609 ± 60.2	759 ± 77.0	536 ± 76.3	668 ± 63.3	495 ± 58.7	521 ± 26.2	488 ± 61.6	463 ± 48.7
*TNFα*	58.6 ± 7.38	102 ± 7.62 **	70.2 ± 8.64	62.4 ± 11.6	59.8 ± 11.9	99.5 ± 13.9 *	62.6 ± 13.9 ^#^	57.9 ± 11.4
Anti-inflammatory								
*IL-10*	106 ± 14.8	113 ± 18.4	169 ± 12.8 *	161 ± 10.3 *	148 ± 17.4	126 ± 13.2	153 ± 15.9	182 ± 15.3
Pleiotropic								
*IL-6*	121 ± 11.9	290 ± 31.9 *	468 ± 93.1 **	311 ± 54.8 *	192 ± 26.8	380 ± 51.7 *	185 ± 51.6 ^#^	270 ± 19.7

## Data Availability

Data are available on request.

## Supplementary Materials

The following supporting information can be downloaded at https://www.mdpi.com/article/10.3390/antiox14030308/s1, Figure S1: Experimental timeline and weekly dosing regimen in relation to hypoglycaemia induction; Figure S2: SFN-mediated improvement in cognitive performance in the NOR and SA tasks was independent of locomotor changes. Table S1: Physiological characteristics of Control, T1D and Nrf2-KO mice following RH and sulforaphane. Table S2: Details of Applied Biosystems TaqMan^®^ gene expression assays used for the real-time PCR analysis.
