# Supplementary material for: Activation of the Nrf2 Pathway by Sulforaphane Improves Hypoglycaemia-Induced Cognitive Impairment in a Rodent Model of Type 1 Diabetes
Source: Antioxidants (Basel). 2025 Mar 4;14(3):308. doi: 10.3390/antiox14030308 (PMC11939732; doi:10.3390/antiox14030308)
Supplement: Supplementary file 1 [file antioxidants-14-00308-s001.zip › antioxidants-3396177-supplementary.pdf]

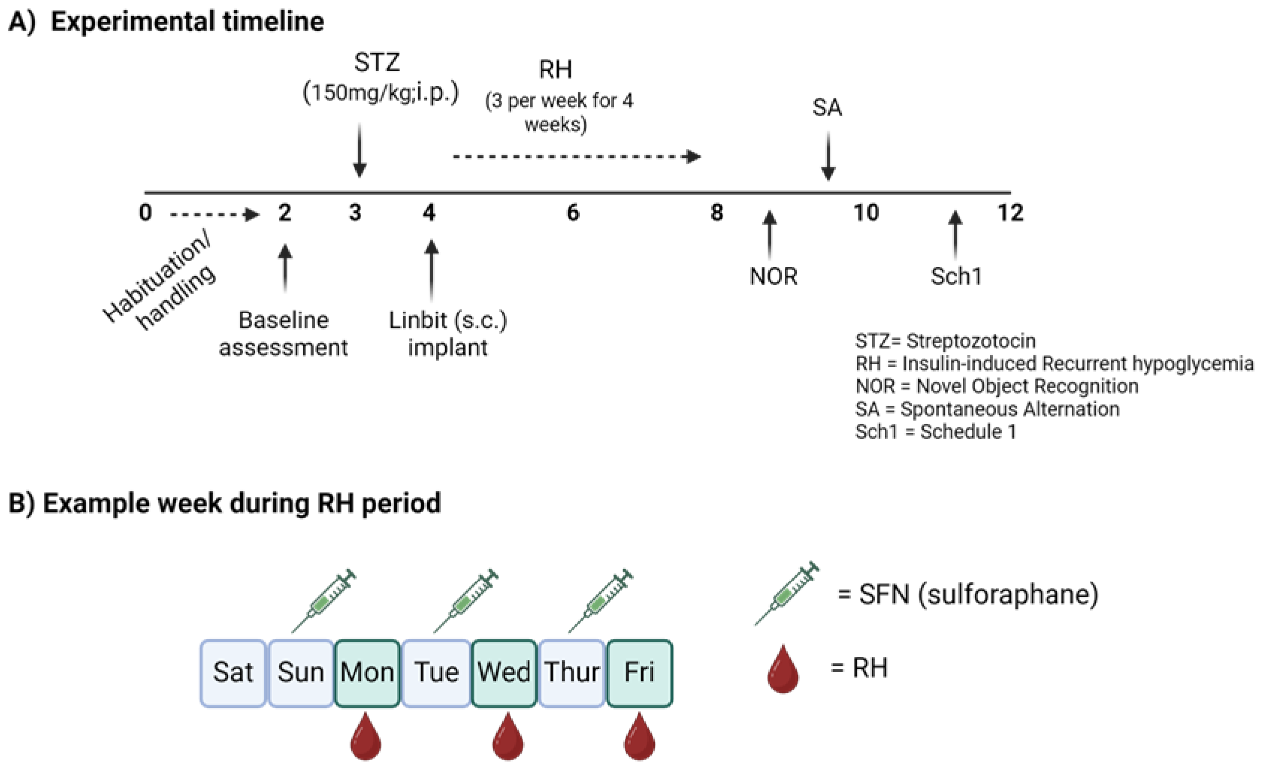

Supplementary Figure S1. Experimental timeline and weekly dosing regimen in relation to hypoglycaemia induction.

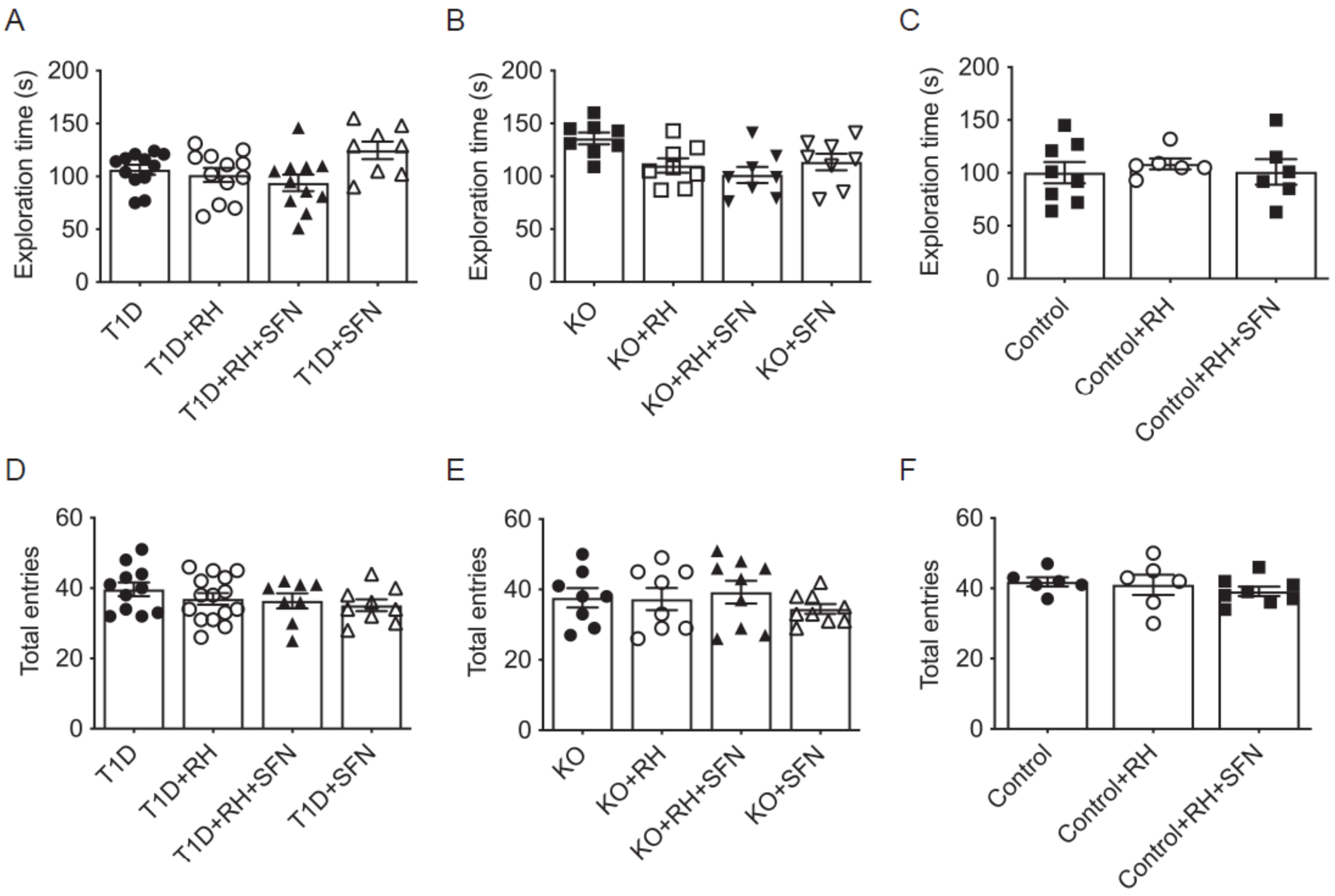

Supplementary Figure S2. SFN-mediated improvement in cognitive performance in the NOR and SA tasks was independent of locomotor changes. RH and sulforaphane did not impact Control, STZ-T1D or Nrf2-KO animals on the total exploration time (A-C) or total arm entries (D-F) in cognitive tests; n = 6-12/group. Results represent the mean values ± SEM. Data were analysed by two-way ANOVA with RH and SFN as between-subject factors, p = ns.

Supplementary Table S1. Physiological characteristics of Control, T1D and *Nrf2*-KO mice following RH and sulforaphane. Body weight change represents the mean difference in body weight between the start and end of the study (pre-STZ) for each mouse group. Blood glucose values are from terminal blood samples (n = 6-12/group). Results represent the mean values ± SEM. * $p < 0.05$, ** $p < 0.01$ vs Control; # $p < 0.05$, ## $p < 0.01$ vs *Nrf2*-KO.

	Weight change (g)	Body weight (g)	Blood glucose (mmol/l)	Mean hypoglycaemia (mmol/l)
**Control**	6.2±1.1#	35.2±1.1	7.1±0.4	NA
**Control + RH**	7.4±1.3	36.4±1.8	7.5±0.4	2.5±0.1
**Control + RH + SFN**	5.7±0.8##	35.1±1.3	6.5±0.4	2.5±0.1
**T1D**	4.3±0.6##	28.8±0.6	20.6±2.0**#	NA
**T1D + RH**	4.1±0.5##	30.3±0.6	22.7±1.5**##	2.8±0.3
**T1D +RH + SFN**	6.3±0.6##	30.2±0.8	22.1±2.5**#	2.9±0.2
**T1D + SFN**	4.3±0.5##	30.5±0.7	19.8±1.7**	NA
**KO**	12.7±1.1*	45.6±1.3	8.9±0.9	NA
**KO + RH**	12.7±2.0**	43.7±2.6	7.9±0.5	2.6±0.2
**KO + RH + SFN**	12.0±1.5	46.8±4.3	8.2±0.6	2.5±0.3
**KO + SFN**	12.5±2.9*	43.6±3.1	7.8±0.8	NA

Supplementary Table S2. Details of Applied Biosystems TaqMan® gene expression assays used for the real-time PCR analysis.

Name	Abbreviation	Assay ID
Glutamate-cysteine ligase catalytic subunit	Gclc	Mm00802655_m1
Glutamate-cysteine ligase modifier subunit	Gclm	Mm01324400_m1
Nuclear factor, erythroid derived 2, like 2	Nfe212	Mm00477784_m1
NAD(P)H dehydrogenase, quinone 1	Nqo1	Mm01253561_m1
Superoxide dismutase 2	Sod2	Mm01313000_m1
Heme oxidase 1	Hmox1	Mm00516005_m1
Actin, beta	Actb	Mm02619580_g1
Cyclophilin A	Pipa	Mm02342430_g1
